# Prevalence and correlates of tobacco use among adolescents in the schools of Kalaiya, Nepal: a cross-sectional questionnaire based study

**DOI:** 10.1186/s12971-016-0075-x

**Published:** 2016-03-31

**Authors:** Ravi Kumar Bhaskar, Mukti Narayan Sah, Kumar Gaurav, Subhadra Chaudhary Bhaskar, Rakesh Singh, Manoj Kumar Yadav, Shatrughna Ojha

**Affiliations:** Department of Community Medicine and Public Health, National Medical College, Birgunj, Nepal; Kalaiya District Hospital, Kalaiya, Bara Nepal; Narayani Sub-Regional Hospital, Birgunj, Nepal

**Keywords:** Adolescents, Determinants, Smoking, Prevalence, Tobacco use, GYTS, Correlates

## Abstract

**Background:**

Adolescent students are vulnerable group for tobacco addiction. Tobacco use among school children is becoming a serious problem in developing countries. This study was carried out to estimate the prevalence of tobacco use and to determine associated factors among adolescent students of Kalaiya municipality.

**Methods:**

A cross sectional survey was carried out by self-administered questionnaire adapted from Global Youth Tobacco Survey to assess tobacco use among the representative sample of 1540 adolescent students selected by stratified random sampling from December 2014 to May 2015.

**Result:**

Overall prevalence of ‘ever users’ of tobacco products was 25.3 %. Prevalence among boys and girls was 31 and 14.4 % respectively. Mean age at initiation of using tobacco was 13.38 ± 1.62 years. The correlates of tobacco use were: sex, ethnicity, family members and friends using tobacco products, and students exposed at home and public place.

**Conclusion:**

School based interventions and tobacco education are necessary to prevent initiation and cessation of tobacco use. Legislations related to tobacco control should be enforced to decrease availability, accessibility and affordability of tobacco products. Social norms of tobacco use among parents and others at home as well as at public place should be modified to curb the tobacco use among school students.

## Background

Tobacco use is a risk factor for six of the eight leading causes of deaths in the world. The six causes are cancers of respiratory tract, ischaemic heart disease, cerebro-vascular disease, chronic obstructive pulmonary disease, tuberculosis and lower respiratory tract infections [1]. Smoking tobacco causes cancer of the lung, larynx, kidney, bladder, stomach, colon, oral cavity and esophagus. It also causes leukaemia, chronic bronchitis, chronic obstructive pulmonary disease, ischaemic heart disease, stroke, miscarriage and premature birth, birth defects and infertility among other diseases [2]. Tobacco use (smoked as well as non-smoked) was also found to be associated with migraine [3].

Tobacco use is the leading global cause of preventable death. Currently about 5 million deaths per year are attributable to tobacco which is expected to rise to more than 8 million deaths a year by 2030. There is estimation that three quarters of these deaths will be in low and middle income countries [2]. Most people begin using tobacco in their adolescent age [4]. Adolescents are the persons in the 10–19 years age group [5]. The use of tobacco among the adolescent and youth, in smoking as well as smokeless forms, is quite high in the South East Asian Region [5]. Nepal is a low income land locked country in SEAR and has 26.6 million populations including 24.2 % adolescents [6].

Geographically Nepal is divided into three Regions: Himal, Pahad and Terai. Himal is the high mountainous area in the northern part of the country. Terai is situated in southern part and it is having plain low land area. Pahad is the hilly area situated in between Himal and Terai. Kalaiya is situated in central Terai region. It is the district headquarter town of Bara district, one out of seventy five districts in Nepal. It has tropical climate. Although majority of the population are Hindus, other religious group includes Muslims, Christians and Buddhists. Kalaiya has multi-ethnic population. There are total 20 schools including 5 government schools.

The Framework Convention on Tobacco control had been signed by Government of Nepal on 3 December 2003 and ratified by parliament on 7 November 2006 [7, 8]. Government had also enforced and implemented the Tobacco Product Control and Regulatory Act 2011, Tobacco Product Control and Regulatory Rule 2012 and Directive for Printing and Labeling of Warning Message and Picture in the Box, Packet, Wrapper, Carton, Parcel and Packaging of Tobacco Product, 2011 in country. This show strong will of government to control tobacco use [7, 8]. Apart from this, prevalence of tobacco use is high as well as it is increasing. Enforcement of tobacco control measures and their weak regulation should be properly implemented to address the problem.

Tobacco is used in a wide variety of ways in Terai (southern plain land of Nepal) including smoking and smokeless form. Tobacco is smoked in the forms of *Beedis, cigarettes* or by using devices like *hooka, chillum* [9]. *Beedi* is a cheap smoking stick, handmade by rolling a dried, rectangular piece of leaf of Diospyros melanaxylon with 0.15–0.25 g of sun-dried, flaked tobacco filled into a conical shape and the roll is secured with a thread. *Hooka* is an indigenous device, made out of wooden and metallic pipes, used for smoking tobacco. *Chilam* is conical pipe made of clay, used for smoking tobacco only or mix of Ganja (Marijuana) and tobacco [9]. Tobacco is used in a number of smokeless forms which include, *Khaini/Surti, gutka, Zarda, snuff* and as an ingredient of *pan masala* [9, 10]. *Khaini/Surti* consists of roasted tobacco flakes which are mixed with slaked lime by keeping on palm of a hand and rubbing it with thumb of another hand and used as a pinch kept in labial or buccal sulcus. *Snuff* is a black-brown powder obtained from tobacco through roasting and pulverization which is used via nasal insufflations. *Gutka* is a manufactured smokeless tobacco product (MSTP), a mixture of areca nut, tobacco and some condiments, marketed in different flavors in colorful pouches. *Pan masala* is a betel quid mixture, which contains areca nut and some condiments, but may or may not contain tobacco. The mixture is chewed and sucked [9].

A national Global Youth Tobacco Survey 2011 (GYTS) in Nepal reported that overall 10 % of the students ever smoked cigarettes and 20.4 % ever used tobacco in other forms [4]. Only 3.1 % students are current smokers while 19.1 % students are current other tobacco users. GYTS 2007 in Nepal revealed that overall 7.9 % of the students ever smoked cigarettes, 8 % used other tobacco product, 3.9 % were current smokers [5]. It reveals tremendous increase in current other tobacco users from 7.2 % in 2001, 8 % in 2007 to 19.1 % in 2011. The trend of use of tobacco is increasing among students [4].

Some of the factors known to be associated with tobacco use among adolescents are age, gender, having smoker friends or parents [5, 11]. Adolescents are vulnerable group for experimenting with tobacco and adopting the risky health behaviour [12]. There is more prevalence of tobacco use among male than among female adolescents [4, 5]. The adolescents having smoking friend or parents are more likely to have tobacco use [12–16].

The present study was carried out with the objectives to estimate the prevalence of tobacco use and to determine the associated factors among adolescent students of Kalaiya municipality of Bara district of central Nepal.

## Methods

### Study design and setting

This was a school based cross-sectional study conducted in Kalaiya Municipality from December 2014 to May 2015. Kalaiya is situated 350 km from capital city, Kathmandu. It is headquarter city of Bara district, which is situated in central Terai region and is bordered in south with Bihar state of India.

### Sample size, study population and sampling technique

The sample size was calculated using formula pq(Z_α_/e)^2^ at 95 % confidence limits and at allowable error 10 % of prevalence [17]. The prevalence of tobacco use was 20.4 % during GYTS 2011 [4]. The calculated sample size was 1499.

Students of grade 8, 9 and 10 from Kalaiya municipality were included in the study. List of schools in Kalaiya was obtained from District Education office. We targeted to distribute at least 100 questionnaires in one school. Out of 20 schools in the list, 4 schools were excluded as they have less than 100 students. Remaining 16 schools were selected and all schools agreed to participate in the study (School response rate = 100 %). To obtain 1499 complete questionnaire, 1649 high school students (assuming 90 % response rate) were targeted for sample selection by random sampling technique.

### Data collection

Data were collected using a self-administered questionnaire adapted from GYTS. The questionnaire was translated into Nepali language and was pre-tested among the adolescent students in different school. Necessary corrections and modifications in the questionnaire were made to make it more comprehensible for the students. Before data collection, an elaborative briefing on the questionnaire was done to all the students of the class of all the selected schools.

A total of 1649 questionnaires were distributed to students in 16 schools and 1550 questionnaires were filled in and submitted to the investigator (student’s response rate = 91.23 %). Ten questionnaires were partly filled, thus they were excluded. Finally 1540 questionnaires were included in analysis.

### Study variables

Tobacco ever use was considered as dependent variable while age, gender, ethnicity, type of school, tobacco use in family members, tobacco use among friends, knowledge about harmful effect, perception about use and exposure to tobacco smoke as explanatory variables.

Ever user was defined as one who had not used any form of tobacco (smoked or chewed) in the past 1 month but had tried in the past. Participants were assessed for ever use of tobacco by asking ‘Prior to the past 30 days, have you ever smoked or chewed tobacco?’ After an affirmative response to this question, Participants were asked about type of tobacco. The options were cigarette, beedi, pan masala, surti, khaini, gutka, snuff, Hookah and specify others, if any.

Current user was defined as one who had used any form of tobacco (smoked or chewed) in the past 1 month. Never user was defined as one who had not tried any form of tobacco. Ethnic groups were broadly classified into Brahmin/Chhetri, Janajati, Dalit and Madhesis as each ethnic group is a collection of many castes which have common customs, socioeconomic, cultural and traditional values. Respondents were asked about harmful effects of tobacco through an open question by asking “can you enumerate the harmful effect of tobacco use. The response was categorized into no knowledge (Reporting no health problems), some knowledge (reporting 1–3 relevant health problems) and good knowledge (Reporting 4 or more relevant health problems). Respondents were asked the responses either yes or no for Tobacco use in family members and tobacco use among their friends. After an affirmative response to this question, Participants were asked about type of tobacco consumed by their family member/friend.

### Data analysis

Data checking, editing and coding was done by the researcher each day. Data entry was done in MS Windows Excel and analysis was done in SPSS 17. Data coding, recording and cleaning was continuously carried out to ensure data quality. Descriptive analysis was done and prevalence was estimated for 95 % confidence interval. Chi-square test was applied to test whether the explanatory variables were significantly associated (*p* value < 0.05) with tobacco use at 95 % confidence interval. Binary logistic regression analysis with backward elimination was used to determine the independence of associations observed in bivariate analysis by controlling for potential confounding factors. Crude and Adjusted Odds ratio (OR/AOR) with 95 % CI were also calculated to quantify the associated risk factors.

### Ethical considerations

Ethical clearance was taken from Institutional Ethical Review Board of National Medical College at the start of the study. Written permission was obtained from selected school authorities. Informed consent was taken from the participants. All the students present at the time of the visit were explained about objectives of the study, were assured of full confidentiality of the responses and asked for their voluntary participation. Absence of any teacher or other personnel at the time of data collection was ensured to prevent response bias.

## Result

### Socio-demographic characteristics

Table 1 shows socio-demographic characteristics of the students. Median age of the respondents was 14.49 ± 2.97 years. Out of total respondents, 65.8 % were male and 34.2 % were female students. Respondents who were Madhesis by ethnicity were predominant. Majority of the respondents were Hindus. Participation was almost equal from government as well as non-governmental school (Ratio = 1:1.24).

**Table 1:** Socio-demographic characteristics of students of kalaiya municipality

Characteristics	Number	Percentage
Age (Years)		
10–12	14	0.9
13–15	1012	65.7
16–18	514	33.4
Grade		
Eight	408	26.5
Nine	777	50.5
Ten	355	23.1
Sex		
Male	1014	65.8
Female	526	34.2
School		
Governmental	854	55.5
Non-governmental	686	44.5
Religion		
Hindu	1299	84.4
Muslim	213	13.8
Others^a^	28	1.8
Ethnicity		
Brahmin/chhetri	213	13.8
Dalits	102	6.6
Janjati^b^	205	13.3
Madhesi^c^	708	46.0
Muslims	213	13.8
Others^d^	99	6.4

^a^ other religion includes Christian, budhism, Jainism

^b^ janjati includes tharu, mandal (dhanuk), magar, limbu

^c^ Madhesi includes Kanu, kalwar, teli, kushwaha, yadav and other terai origin castes

^d^ other ethnicity includes marwadi, Bengali, tamils

Some students reported initiation of tobacco use as early as 6 years and maximum age of initiation was 16 years. The mean age of initiation was 13.38 ± 1.62 years.

### Prevalence of ever and current tobacco use

The prevalence of ever tobacco use was 25.3 % in our study. The prevalence of ever tobacco use among male and female students was 31 % and 14.4 % respectively. Among the total ever tobacco users (Prevalence = 25.3 %), 7.7 % had smoked (Cigarrette/Beedi), 9.2 % had used Surti/Khaini and 8.4 % had used Gutka/pan masala (Table 2).

**Table 2:** Prevalence of tobacco use among students

Charecteristics		Prevalence of ever use of tobacco in any form	Prevalence of ever use by type of tobacco			Never user of tobacco in any form
			Cigarette/beedi	Surti/khaini	Gutka/Pan-masala	
Total		25.3 %	7.7 %	9.2 %	8.4 %	74.7 %
Sex	Male	31 %	9.4 % (	10.1 %	10.7 %	69 %
	Female	14.4 %	4.3 %	4.3 %	5.7 %	85.6 %
Type of school	Government school	25.1 %	5.4 %	9.3 %	10.4 %	74.9 %
	Non-government school	25.7 %	11.2 %	7.4 %	7.1 %	74.3 %
Religion	Hindu	24.4 %	7.3 %	8.6 %	8.5 %	75.6 %
	Muslim	31 %	9.9 %	12.7 %	8.4 %	69 %
	Others^a^	21.4 %	7.1 %	10.7 %	3.6 %	78.6 %
Ethnicity	Brahmin/chhetri	10.3 %	4.2 %	3.8 %	2.3 %	89.7 %
	Dalits	36.3 %	12.7 %	11.8 %	11.8 %	63.7 %
	Janjati^b^	41.5 %	15.6 %	15.1 %	10.7 %	58.5 %
	Madhesi^c^	21.6 %	5.2 %	7.8 %	8.6 %	78.4 %
	muslim	31 %	9.9 %	12.7 %	8.5 %	69 %
	Others^d^	26.3 %	6.1 %	9.1 %	11.1 %	73.7 %

^a^ other religion includes Christian, budhism, Jainism

^b^ janjati includes tharu, mandal (dhanuk), magar, limbu

^c^ Madhesi includes Kanu, kalwar, teli, kushwaha, yadav and other terai origin castes

^d^ other ethnicity includes marwadi, Bengali, tamils

### Bivariate analysis

In the bivariate analysis, all the explanatory variables except type of school was found to be significantly associated with ever use of tobacco. Male students were more likely to ever use of tobacco than the female students (OR = 2.65;95%CI: 2.01–3.50). Compared to Brahmin/Chhetri students, Janjatis were six times more likely to ever use of tobacco (OR = 6.15; 95 % CI: 3.65–10.36). Muslim students had nearly four times the odds of ever using tobacco than Brahmin/Chhetri students (OR = 3.90; 95 % CI: 2.30–6.61). students whose family members were using tobacco were more likely to ever use tobacco than the students whose family members were not using tobacco (OR = 11.07; 95 % CI: 7.71–15.9). Students having tobacco user friends were nearly 15 times more likely to ever use tobacco than the students having tobacco non-user friends (OR = 14.82; 95 % CI: 10.78–20.36) (Table 3).

**Table 3:** Different characteristics of respondents and tobacco use (bivariate analysis)

Characteristics	Ever user *n* (%)^a^	Non-user *n* (%)^a^	*p*-value	Crude OR (95 % CI)
Sex				
Boys	314 (80.5)	700 (60.9)	<0.01	2.65 (2.01–3.50)
Girls	76 (19.5)	450 (39.1)		1
Schools				
Government	214 (54.9)	640 (55.7)	0.789	0.97 (0.77–1.22)
Non-government	176 (45.1)	510 (44.3)		1
Ethnicity				
Brahmin/chhetri	22 (5.6)	191 (16.6)	<0.01	1
Dalits	37 (9.5)	65 (5.6)		4.94 (2.72–8.99)
Janjati	85 (21.8)	120 (10.4)		6.15 (3.65–10.36)
Madhesi	154 (39.5)	554 (48.2)		2.41 (1.49–3.88)
Muslim	66 (16.9)	147 (12.8)		3.90 (2.30–6.61)
Others	26 (6.7)	73 (6.3)		3.10 (1.65–5.80)
Knowledge about harmful effect of tobacco use				
No knowledge	128 (32.8)	238 (20.7)	<0.01	3.37 (2.53–4.5)
Some knowledge	149 (38.2)	153 (13.3)		6.11 (4.54–8.21)
Good knowledge	121 (31.0)	759 (66.0)		1
Family members using tobacco				
Yes	354 (90.8)	541 (47.0)	<0.01	11.07 (7.71–15.9)
No	36 (9.2)	609 (53.0)		1
Friends using tobacco				
Yes	338 (86.7)	350 (30.4)	<0.01	14.82 (10.78–20.36)
No	52 (13.3)	800 (69.6)		1
Exposed at home				
Yes	330 (84.6)	300 (26.1)	<0.01	15.58 (11.49–21.14)
No	60 (15.4)	850 (73.9)		1
Exposed at Public place				
Yes	374 (95.9)	986 (85.7)	<0.01	3.89 (2.30–6.59)
No	16 (4.1)	164 (14.3)		1

^a^Indicates percentage of column

### Multivariate analysis

After multivariate analysis, Male students were more likely to be ever tobacco users compared with female students (AOR = 3.2; 95 % CI: 1.4–5.2). Students from Janajati ethnicity were more than five times likely to be ever users of tobacco than those who were Brahmin/Chhetris (AOR = 5.41, 95 % CI: 2.98–7.63). The adolescents who were exposed to smoking at home had higher odds of ever using tobacco compared with those who were not exposed (AOR = 12.12; 95 %:1.69–86.82). Students whose family members used tobacco were twenty times more likely to be ever tobacco user than the students whose family members did not use tobacco (AOR = 20.16; 95 % CI: 1.92–210.30) (Table 4).

**Table 4:** Association of different variables with tobacco use among adolescent students (Multivariate analysis)

Characteristics	Adjusted Odds ratio (95 % CI)	*p*-value
Sex		
Boys	3.21 (1.42–5.27)	<0.05
Girls	1.0	
Schools		
Government	1.0	0.29
Non-government	0.30 (0.03–2.77)	
Ethnicity		
Brahmin/chhetri	1	<0.05
Dalits	1.78 (1.03–2.17)	
Janjati	5.41 (2.98–7.63)	
Madhesi	1.55 (0.89–3.12)	
Muslims	2.31 (1.34–4.65)	
Others	1.98 (0.98–3.34)	
Knowledge about harmful effect of tobacco use		
No knowledge	6.17 (0.44–84.92)	0.17
Some knowledge	5.44 (0.78–29.66)	
Good knowledge	1	
Family members using tobacco		
Yes	20.16 (1.93–210.30)	<0.05
No	1	
Friends using tobacco		
Yes	3.78 (1.93–17.85)	<0.05
No	1	
Exposed at home		
Yes	12.12 (1.69–86.82)	<0.05
No	1	
Exposed at Public place		
Yes	1.39 (0.05–3.74)	.24
No	1	

## Discussion

Tobacco use is one of the serious and death-causing public health problems all over the world. This study aiming to assess prevalence and factors associated with tobacco ever use among adolescents has described the magnitude and associated factors which have been discussed as follows.

The prevalence of ever use of tobacco in this study was 25.3 % which was more comparable with the findings of a study from Iraq [12]. But it was high compared with a national survey and other studies from Nepal and India [4–7, 9–12, 17]. Some studies reported high prevalence of tobacco use than this study [6, 7, 9–15, 17]. The trend of prevalence of ever use of tobacco seems to be increasing from 7.8 % in 2001, 9.4 % in 2007, 13.9 % in 2008, 20.4 % in 2011 to 25.3 % in this study [4, 7–11, 13–15, 17, 18].

The prevalence of ever as well as current use of tobacco among male students was found more compared to the female students. This finding is comparable with other studies [4, 5, 7, 12].

The prevalence of ever smoker was found 7.7 % in this study which is more in accordance with a national survey [8]. But some other studies show higher prevalence of ever smoker than this study [4, 5, 16, 19, 20]. The prevalence of current tobacco use was 19.5 % in this study. It is more comparable to GYTS 2011 but it is less than the findings of a study from east Nepal [4, 5].

The mean age for tobacco use initiation (smoking and chewing) in our study was found to be in consistency with studies from Dharan, Kathmandu, Noida and Kerala where the mean ages of onset were 13.79, 14.15, 12.4 and 13.2 years, respectively [4–22].

Sex was found significantly associated with ever tobacco use. There were higher odds of tobacco use among boys as compared with girls. Similar finding was seen in other studies conducted in Nepal and abroad [5, 7, 12, 14, 23]. Ethnicity was significantly associated with ever tobacco use. Tobacco ever use was more likely to exist among Janjatis and Dalits, compared to Brahmins/Chhetris. This finding is comparable with other study [5]. Lower socio-economic status and prevalent illiteracy among Janjatis and Dalits may have some influence on tobacco use among the group. Among Janjatis and Dalits, tobacco use may be culturally acceptable while among Brahmin and Chhetri family, it is considered as forbidden.”

Knowledge regarding harmful effect and health hazard of tobacco use was found significantly associated with ever use of tobacco in other studies [7–11, 13–27]. Bivariate analysis revealed the same finding in this study. But after adjustment for the confounders, result was not in concordance with them. Students whose family members were tobacco users had higher risk of tobacco use relative to those with no family members using tobacco. This finding seems to be consistent with other studies [5–7, 9–13, 16, 17]. Students having friends using tobacco were at more risk of ever using tobacco than the students having no friends using tobacco. The same result was seen in other studies [12–16]. Friend’s tobacco consumption was found to be a significant link between peer pressure and tobacco use [18].

The present study had few limitations. Even though the participation in the study was completely voluntary with a declaration of non-disclosure of identity and confidentiality, chances of bias may occur in the findings as the data were collected through self-administered questionnaire. The assessment of the tobacco-use status was based entirely upon the response given by the subject believing that false reporting was very unlikely. However, this was not validated by biomarkers. Sample size of the study was small and limited to school-going adolescents of Kalaiya only, hence cannot be generalised. The temporal association between the independent variables and tobacco use could not be established due to cross-sectional nature of the study.

## Conclusion

Tobacco use was prevalent among the adolescent students despite the existence of anti-tobacco regulations in the country. Male gender, Janajati, Dalits and Muslims by ethnicity, students having family members and friends using tobacco, students exposed to tobacco at home or at public place were significantly at risk of ever using tobacco. School based interventions and tobacco education are necessary to prevent initiation and cessation of tobacco use. Legislations related to tobacco control should be enforced to decrease availability, accessibility and affordability of tobacco products. Social norms of tobacco use among parents and others at home as well as at public place should be modified to curb the tobacco use among school students.

Further researches are needed to explore the vulnerability of certain ethnic groups towards tobacco use to generate an effective awareness campaign.
